# Critique of the pairwise method for estimating qPCR amplification efficiency: beware of correlated data!

**DOI:** 10.1186/s12859-020-03604-4

**Published:** 2020-07-08

**Authors:** Joel Tellinghuisen

**Affiliations:** Department of Chemistry, Vanderbilt University, Nashville, TN 37235 USA

**Keywords:** qPCR, Amplification efficiency, Data analysis, Weighted least squares, Statistical errors, Calibration, Correlated data

## Abstract

**Background:**

A recently proposed method for estimating qPCR amplification efficiency *E* analyzes fluorescence intensity ratios from pairs of points deemed to lie in the exponential growth region on the amplification curves for all reactions in a dilution series. This method suffers from a serious problem: The resulting ratios are highly correlated, as they involve multiple use of the raw data, for example, yielding ~ 250 *E* estimates from ~ 25 intensity readings. The resulting statistics for such estimates are falsely optimistic in their assessment of the estimation precision.

**Results:**

Monte Carlo simulations confirm that the correlated pairs method yields precision estimates that are better than actual by a factor of two or more. This result is further supported by estimating *E* by both pairwise and *C*_*q*_ calibration methods for the 16 replicate datasets from the critiqued work, and then comparing the ensemble statistics for these methods.

**Conclusion:**

Contrary to assertions in the proposing work, the pairwise method does *not* yield E estimates a factor of 2 more precise than estimates from *C*_*q*_ calibration fitting (the standard curve method). On the other hand, the statistically correct direct fit of the data to the model behind the pairwise method *can* yield *E* estimates of comparable precision. Ways in which the approach might be improved are discussed briefly.

## Background

The goal of quantitative polymerase chain reaction (qPCR) is the quantification of small amounts of a targeted genetic material, either relative to a chosen reference substance [1, 2] or absolute [3–5]. The standard approach for the latter is the use of a calibration series obtained by amplifying a set of solutions containing the targeted species at known concentrations designed to encompass that of the unknown. The resulting data — usually fluorescence signal as a function of amplification cycle — are then analyzed to obtain a characteristic cycle marker called *C*_*q*_ (quantification cycle), which can be defined in several ways, including threshold signal (either absolute or relative) and first- and second-derivative maxima [6–8]. Under the assumption that *C*_*q*_ falls in the region of exponential growth,

1 $$ y={y}_0{E}^x, $$

— where *y*_0_ is the fluorescence signal for *N*_0_ target molecules in cycle *x* = 0, and *E* is the amplification efficiency (1 ≤ *E* ≤ 2) — a plot of *C*_*q*_ vs log (*N*_0_) is linear, with slope = − 1/log(*E*). The location of *C*_*q*_ for the unknown on this line thus determines its *N*_0_.

To achieve adequate precision in such calibration (or standard curve) procedures, it is generally recommended to record the known curves in replicate, typically triplicate. That generally means 12–18 reactions (and ideally a comparable number of reactions for the unknown, but this is seldom followed). As an alternative to this data-intensive procedure, many workers have sought what might be considered the “holy grail” of qPCR — the estimation of *N*_0_ from just the curve(s) for the unknown [8]. This requires reliable estimation of *E*, which must be based on analysis of just the early growth region, because *E* declines to ~ 1 as the signal plateaus in the large-*x* limit. If trustworthy *E* estimates can be obtained, then only a single calibration result is needed to relate *y*_0_ to *N*_0_ for the unknown, or not even that if information relating fluorescence signal to amplicon size can be trusted [9]. However, for most methods used to estimate *E* for single reactions (SR), the results are likely to be low-biased, since *E* has already declined by the time the signal has risen above background sufficiently to permit its estimation [10].

**Fig. 1:**
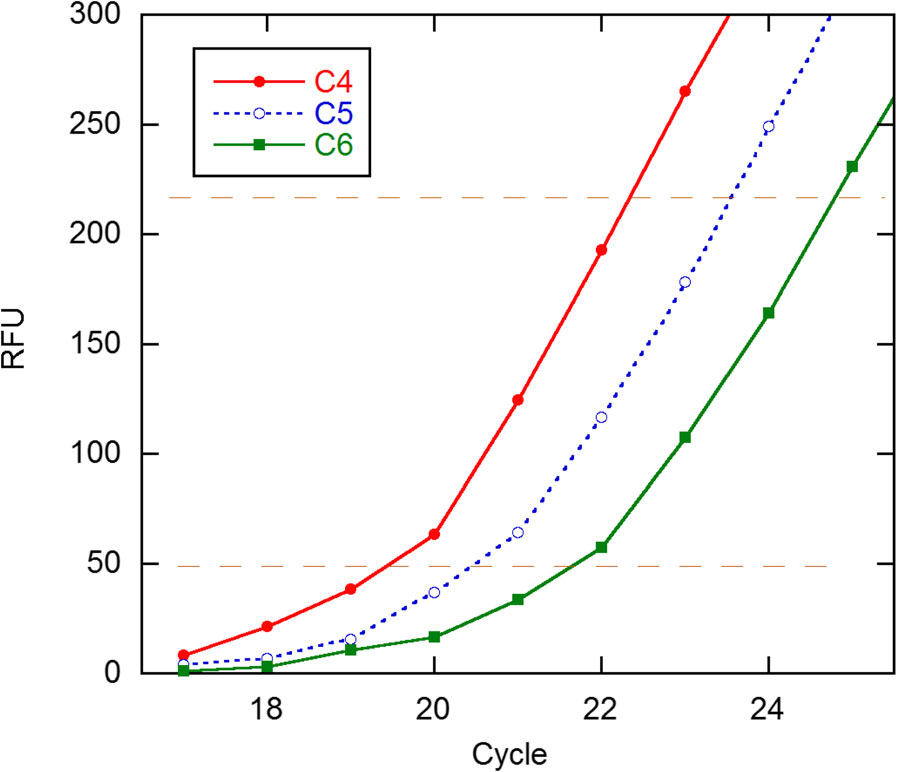
qPCR fluorescence data displayed in Fig. 2a in ref. [11]. The dilution factor is 2 for these curves, which represent the lowest 3 concentrations in the first C series. The horizontal dashed lines demarcate the exponential growth zone; this was set lower — 20-180 — for the full analysis in [11]. The RFU (relative fluorescence units) plateau values are ~ 600

Recently Panina, et al. (PGDW) have proposed a method for estimating *E* that might be considered an intermediate to the traditional *C*_*q*_ calibration method and the SR methods [11]. In it, signal values deemed to lie in the early growth phase on all the amplification curves in a dilution series are analyzed simultaneously to yield a single estimate of *E*. This “global” procedure is illustrated in their Fig. 2, part (a) of which is reproduced here in Fig. 1. The dilution factor here is 2, so the 9 points to be analyzed can be fitted to the two parameters *y*_0_ and *E* using.

2 $$ {y}_{ij}={y}_0{E}^{x_{ij}}/{2}^j, $$

where *y*_0_ refers to the most concentrated solution (leftmost curve, *j* = 0), *x*_*ij*_ is the *i*th cycle on the *j*th curve, and the data are assumed to be background-free (baselined). Rather than do this, PGDW analyzed pairs of values, which might be fitted to.

3 $$ {\mathrm{y}}_{\mathrm{ij}}-{y}_{kl}={y}_0\left({E}^{x_{ij}}/{2}^j-{E}^{x_{kl}}/{2}^l\right), $$

In this case there would now be $$ \left(\begin{array}{c}9\\ {}2\end{array}\right) $$ = 36 pairs. However, PGDW chose to take ratios to eliminate *y*_0_, estimating *E* from

4 $$ {\log}_2\left({E}_{ij}\right)=\left[{\log}_2\left({y}_j\right)-{\log}_2\left({y}_i\right)+D2-D1\right]/\left(j-i\right), $$

where *y*_*j*_ and *y*_*i*_ are the signals of the pair, having cycle numbers *x* = *j* and *i*, respectively, and *D*2 and *D*1 are the dilution factors (powers of 2). Because of the factor in the denominator, pairs having the same cycle number must be excluded, reducing the number of pairs in this case from 36 to 31.

There is a fundamental statistical problem with this pairwise approach: The 36 (31) data values are not *independent*, rather are correlated, since many of them involve the same members of the initial 9 points, which *are* reasonably assumed to be independent. The problem here might be recognized more easily through the following example: Suppose we just want the average of 9 values. Recognizing that the standard error (SE) (standard deviation in the mean in this case) is proportional to $1/\sqrt{n}$, why not just take each value 4 times and thereby reduce the SE by a factor of 2? This might seem silly, but the use of pairwise differences or ratios is just a more complex version of the same idea. In short, the full information in the data set is contained in the original 9 points, and there is no way of manufacturing more information through any scheme that generates more apparent observations. Since most of the statistical tests employed by PGDW assume independent data, they too are unreliable when applied to these pairwise estimates.

qPCR fluorescence data are expected to have roughly constant noise in the baseline and early growth regions [10], and PGDW found this for their data. Calling this random data error *σ*_*y*_, fits to Eq. 2 can properly use unweighted nonlinear LS (NLS). For the differences in Eq. 3, the same would hold for independent data, but now with variance = 2*σ*_*y*_^2^ from error propagation [12]. Calculations for the 36-differences model give a predicted SE for *E* smaller than that for the direct 9-point model by a factor of 1.9, close to the factor 2 for the averages analogy given above. Again, this is for independent difference values, so it represents the *apparent* precision gain, but will *not* hold for the correlated actual differences.

The *E*_*ij*_ estimates from Eq. 4 have varying precision, requiring weighting for the averages and normalized residuals for assessing outliers. Neglecting this can drastically affect both the residuals analysis and the normality tests, even apart from the correlation problems. To get the weights, we apply error propagation to a modified version of Eq. 4,

5 $$ Z\equiv 1\mathrm{n}\left({E}_{ij}\right)=\left[1\mathrm{n}\left({y}_j\right)-1\mathrm{n}\left({y}_i\right)+\left(D2-D1\right)1\mathrm{n}2\right]/\left(j-i\right), $$

6 $$ \mathrm{obtaining}\kern0.5em {\sigma}_z^2={\left(\frac{\partial Z}{\partial {y}_j}\right)}^2{\sigma}_y^2+{\left(\frac{\partial Z}{\partial {y}_i}\right)}^2{\sigma}_y^2=\frac{\sigma_y^2}{{\left(j-i\right)}^2}\left(\frac{1}{y_j^2}+\frac{1}{y_i^2}\right) $$

The weights *w*_*ij*_ for averaging the *E*_*ij*_ are proportional to ${\sigma}_{E_{ij}}^{-2}$, with *σ*_*Z*_ = *σ*_*E*_/*E*. Recognizing that the *E* estimates are narrowly distributed, this gives approximately

7 $$ {w}_{ij}\propto \frac{{\left(j-i\right)}^2{y}_i^2{y}_j^2}{y_i^2+{y}_j^2} $$

which heavily favors ratios obtained for large cycle differences. The normalized residuals are obtained by dividing $\left({E}_{ij}-\overline{E}\right)$ by ${\sigma}_{E_{ij}}$.

These statistical flaws do not necessarily mean that the method of PGDW is of no value, just that the resulting statistics cannot be trusted. One test that compares the methods on equal footing is their performance in ensemble statistics, which can be done here, thanks to the 16 replicate 6-concentration datasets collected by PGDW. Such ensemble-based statistics remain valid, though limited in precision for just 16 replicates. However, if the pairwise method is to be employed, its proper (and much easier) implementation is through direct fitting to Eq. 2. In what follows, I use Monte Carlo simulations to confirm that this direct fitting approach is better than the pairwise method. I also find that the *C*_*q*_ calibration results in PGDW are not optimal, rather can be improved significantly through better methods of estimating *C*_*q*_ [7]. Further, pairwise *E* estimates tend to be low-biased for the same reason as found in [10].

## Results

**Fig. 2:**
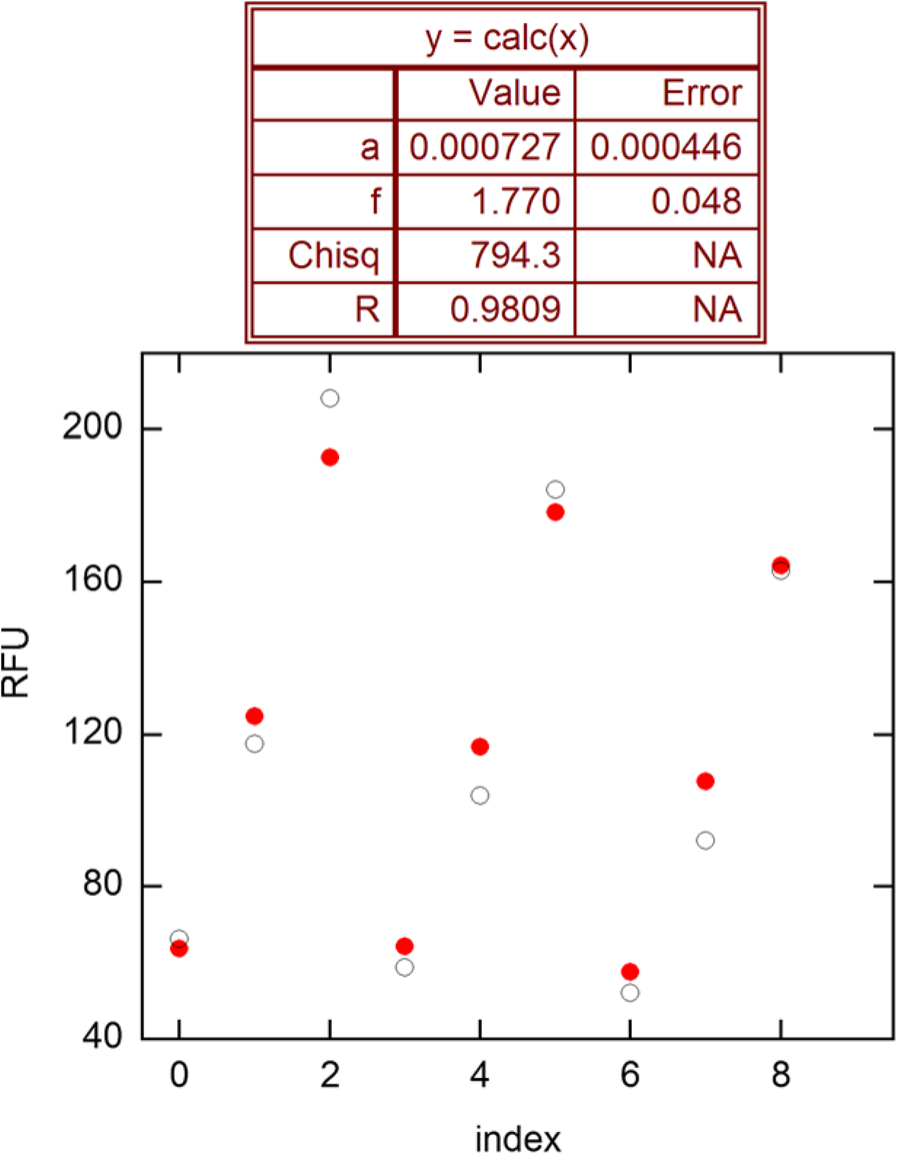
Fit of the growth-zone points in Fig. 1 (solid) to the model of Eq. 2. Open points are calculated values. “Error” is the estimated SE. Chisq is the sum of squared residuals, from which the estimated variance is *s*_*y*_^2^ = 794.3/7 = 113.5, giving *s*_*y*_ = 10.7

Figure 2 shows results obtained by fitting the data in Fig. 1 to the model of Eq. 2. The residuals are systematic, leading to an estimate of 10.7 for the data error. This systematic error becomes even clearer if the cycles are all dropped by 1 unit, giving Chisq = 40.83 (*s*_*y*_ = 2.4) and *E* = 1.854(19) (where figures in parentheses are in terms of final digits, meaning 1.854 ± 0.019 here). Alternatively, 12 points fall in the growth zone 20–180 adopted by PGDW and give *E* = 1.747(27) and *s*_*y*_ = 6.7. These results show the sensitivity to the selection of the growth zone and are consistent with a main finding of ref. [10], that when growth has proceeded far enough above background to be analyzed, *E* is already declining significantly.

**Fig. 3:**
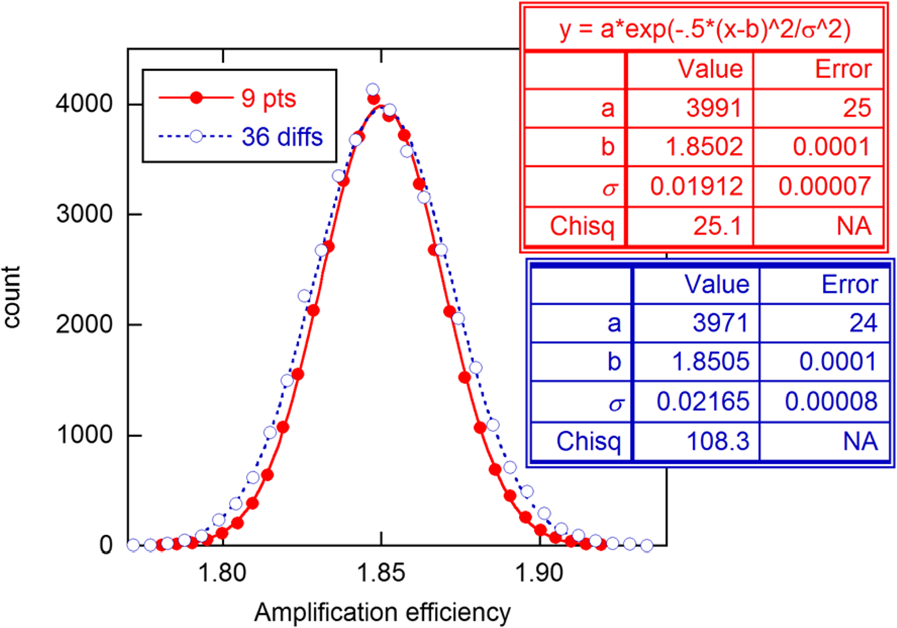
Histogrammed *E* values from 4 × 10^4^ simulations of the 9-point model described in text, fitted directly to Eq. 2 and converted to differences for fitting to Eq. 3. The boxes show the results from fitting to the normal distribution, in which the counts were weighted inversely as the count (Poisson approximation)

Monte Carlo simulations were done on a 9-point model resembling the second mentioned above, having *E* = 1.85 and *σ*_*y*_ = 2.4. For each generated data set, random normal error of magnitude *σ*_*y*_ was added to the 9 exact intensity values of the model, and these randomized data were then fitted directly to Eq. 2. Next the 36 differences were calculated and analyzed with the model of Eq. 3. Histogrammed results for *E* are shown in Fig. 3, with fits of the counts to the normal distribution. The Chisq (*χ*^2^) value is consistent with expectations for the *ν*  = 27 statistical degrees of freedom in the case of the 9-point model, but greatly exceeds *ν* (28) for the pairs, showing that the *E* estimates from the pairs analysis are not normally distributed [13]. The *σ* value for the 9-point model agrees with predictions, while that for the pairs exceeds predictions for *independent* data by more than a factor of 2 and the 9-point results by 13%. Results for the ratios approach of Eq. 4 used by PGDW are similar to those for differences (see Supplementary Information, SI). These findings show that the parametric SEs and set-by-by set statistics from pairs analysis greatly overestimate precision, while the actual precision is nominally worse than for the simpler direct analysis.

**Fig. 4:**
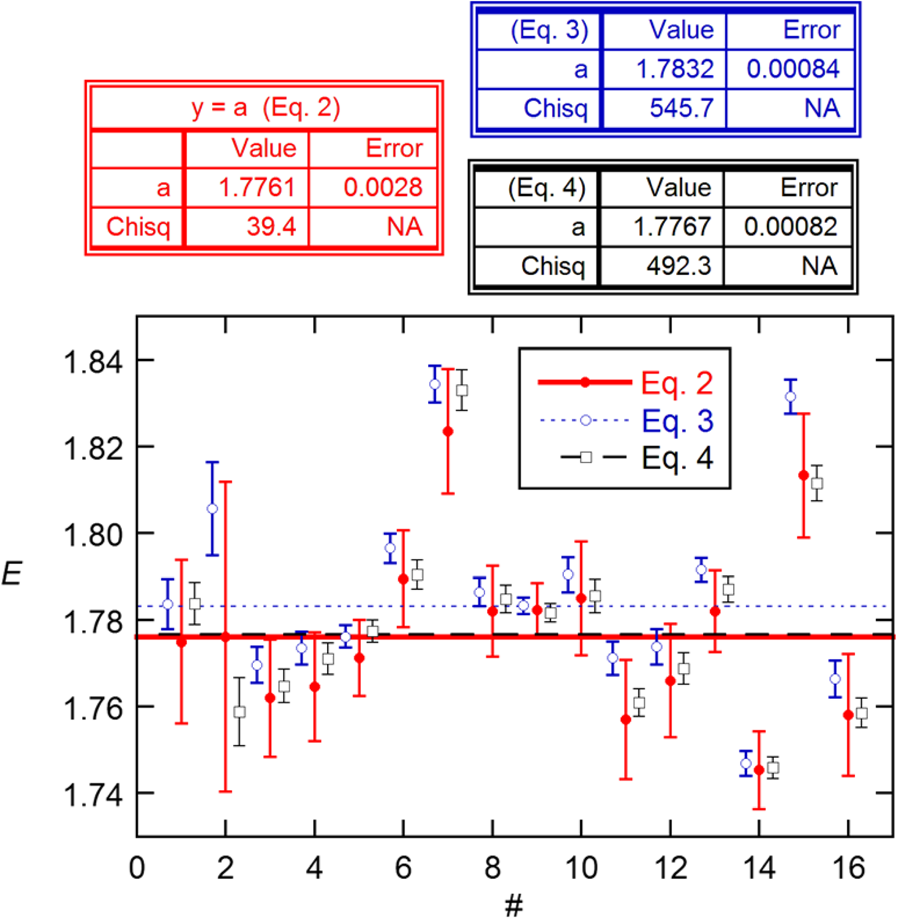
*E* estimates obtained analyzing the 16 6-replicate data sets from PGDW using Eqs. 2–4, and their averages (lines). The *x*-axis numbers represent the data sets, as A1–6, A7–12, …, H7–12, with points for Eqs. 3 and 4 displaced slightly for display purposes. The KaleidaGraph fit results include a priori SEs (Error) [14]. The post-SEs are these values × (*χ*^2^/*ν*)^1/2^ = 0.0045, 0.0051, and 0.0047

All 16 6-reaction replicate sets from PGDW were analyzed by all three of these approaches, using the 20–180 RFU growth window employed by PGDW. The results (Fig. 4) follow expectations from the results of the MC simulations. The numbers of points range from 22 to 25, resulting in 231–300 differences analyzed by Eq. 3 and 211–277 by Eq. 4. The large number of differences leads to the vastly optimistic error bars and the excessive Chisq values (cf *ν* = 15) for these methods. The post-SEs from these weighted means can be converted to effective SDs for each replicate set by multiplying by $\sqrt{16}$, giving 0.0180, 0.0204, and 0.0189. Unweighted averaging gives 0.0201, 0.0228, and 0.0215. Since sampling estimates of SDs have relative SD = (2*ν*)^-1/2^(18%), all of these SDs can be considered statistically consistent. They are also all about a factor of 2 larger than reported by PGDW.

**Fig. 5:**
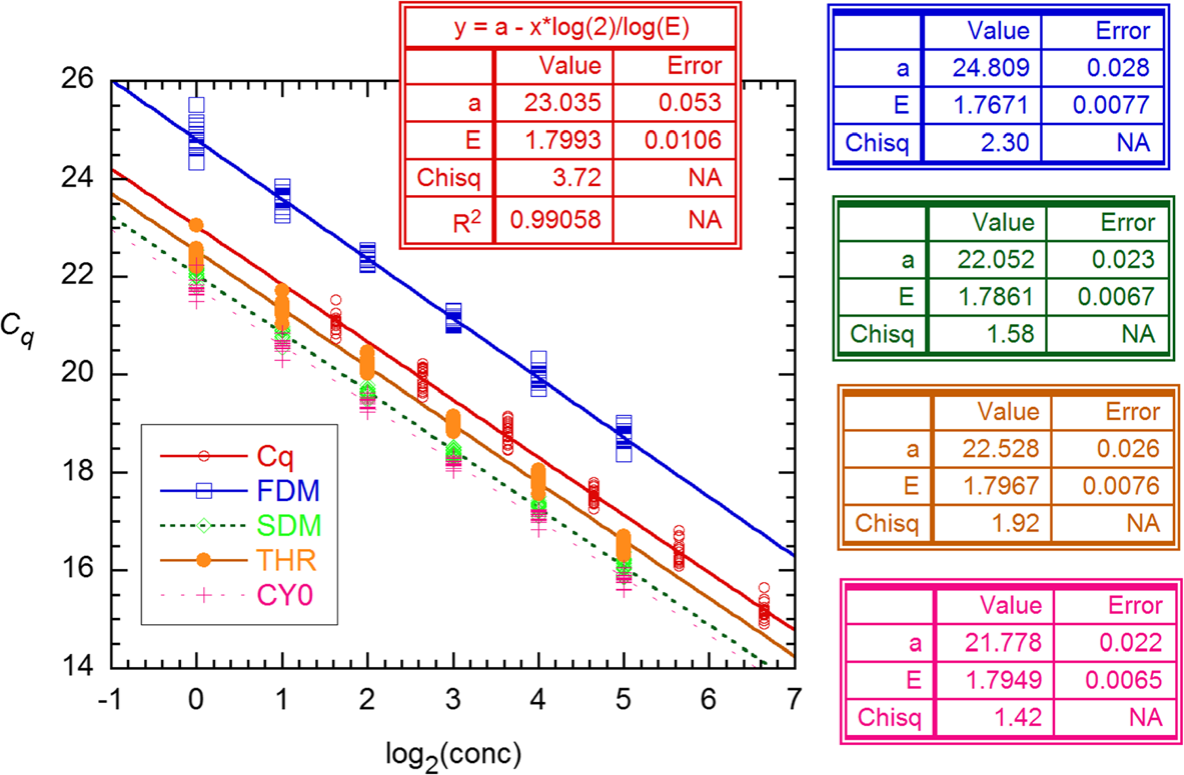
LS fits of all 96 *C*_*q*_ values from [11] and those obtained for 4 different *C*_*q*_ markers using the methods of ref. [7]. The *χ*^2^ values (Chisq) are a direct measure of the precision of the data and indicate that the Cy0 estimates are best [15]

**Fig. 6:**
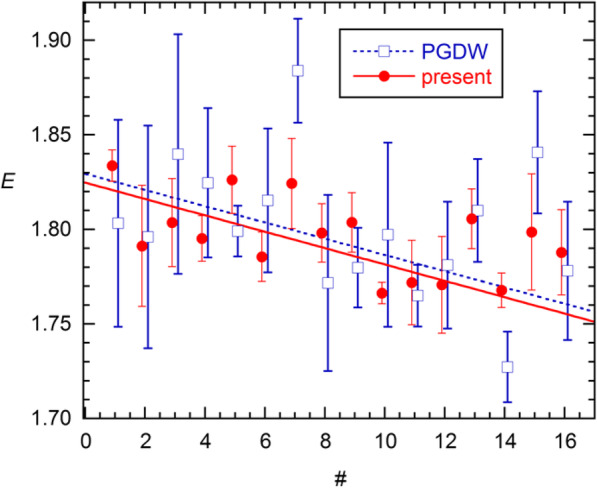
Calibration-based *E* estimates for the 16 6-point replicate datasets from [11], from *C*_*q*_ values reported by PGDW and from Cy0 values obtained using methods of [7]. The lines are results from weighted LS fits; the slope is − 0.0043(7) from the present results

Before addressing this difference, I consider the other method of assessing the AE: calibration (standard curve) fitting of *C*_*q*_ vs. log(*N*_0_). Figure 5 shows such fits of all *C*_*q*_s reported by PGDW and those estimated from their data using the methods of ref. [7]. All of the latter gave *χ*^2^ values smaller than those from PGDW, with lowest for Cy0. The SI describes the procedures from [7] in detail, including modifications that gave even better Cy0 values (*χ*^2^ = 1.27). These were used to generate 6-point standard curves for each of the 16 replicate sets. The *E* estimates from these are displayed in Fig. 6, where they show a clear decline with increasing dataset number, information not evident from the results in Fig. 4. Although the slope is statistically significant, it must be an artifact of the experiments. Accordingly, simple averaging yields *E* = 1.8008(91) for PGDW and 1.7956(52) for my results; the single-set SDs are larger by the factor 4, giving 0.0363 and 0.0207. The latter value agrees with results given earlier for analysis with Eqs. 2–4.

**Fig. 7:**
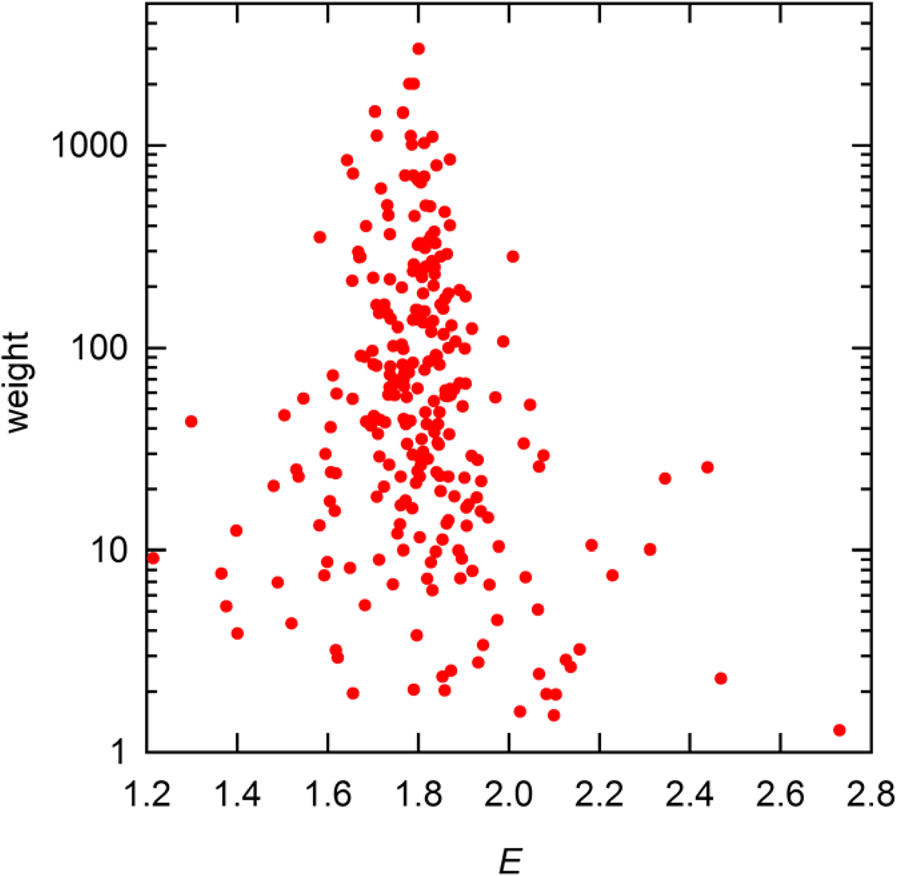
Statistical weights for pairwise *E* estimates from the A1–6 dataset, displayed as a function of *E*. A single value at *E* = 1.58 has *w*_*i*_ = 351; all others in the outlier zone (*E* < 1.60 and *E* > 2.15) have *w*_*i*_ < 60. The full range of *w*_*i*_ is 1.3–3000

I turn now to a closer examination of the pairwise results of PGDW. The numbers of ratios analyzed by my routine are close to the numbers reported in their Table S8, but not identical. For example, for the first 4 replicate sets, my analysis included 254, 254, 257, and 256 values, while they report 258, 258, 244, and 241. PGDW deleted ~ 15 values from each set, judging them to be outliers. However, as shown in Fig. 7, with proper weighting, these values are *ipso facto* insignificant. Thus, the values in the designated outlier zone have average weight two orders of magnitude smaller than the overall maximum weight. I have examined the distributions of weighted residuals by binning these and fitting the resulting histograms to the normal distribution. Viewed collectively, the 7 sets I have analyzed are not convincingly normal, which is not surprising, considering the correlated nature of the data. Still, they are not radically nonnormal, as the *χ*^2^ values of these fits are consistent with probabilities ranging from 1 to 50% (see Supplementary information).

Comparison of my *E* estimates with those in Table S7 from [11] shows general agreement, except for the additional values from my routine. Thus, the statistical properties of my estimates must be essentially the same as those of PGDW. With proper weighting, the estimates for each replicate set appear more precise than without. For example, weighted averaging for the A1–6 set gives *E* = 1.7838(48), while unweighted averaging gives 1.8070(106), where the errors are the SEs. These would be the correct quantities for predicting the precision of estimation for the method, if the data were independent; and they appear to be what PGDW obtained from their MC resampling method. However, as already noted, these SEs are falsely optimistic because of the correlated data problem.

## Conclusion

A detailed examination of the pairwise method of estimating AE from Panina, et al. [11] shows that the appearance of improved precision for the method is a spurious result of the inherent correlation of the data. When implemented in a statistically correct way, by direct fitting of the fluorescence data to Eq. 2, the method can yield *E* estimates of precision comparable to that obtained by the standard curve approach. However, in the present tests, the latter revealed a trend toward smaller *E* with the increasing number of the replicate dataset, a trend not evident from the Eq. 2 analysis.

Comparing the average *E* from *C*_*q*_ calibration [1.7956(52)] with that from Eq. 2 in Fig. 4 [1.7761(45)], we see that the latter is about two combined SEs lower than the former, thus marginally biased. It is possible that the simple exponential growth assumption in Eq. 2 could be replaced by a more realistic growth model, like the logistic model [16, 17],

8 $$ y(x)=\frac{y_0{y}_{\mathrm{max}}{E}_0^x}{y_0{E}_0^x+{y}_{\mathrm{max}}-{y}_0} $$

in which *E* declines naturally in the growth region from its value *E*_0_ in the baseline region. This might permit expansion of the range of fitted points with little bias.

The case against the pairwise method presented here has been made partly on its performance in comparisons of ensemble statistics. In fact it is not unusual for the latter to show somewhat larger dispersion than expected on the basis of statistics for the individual values. Thus, e.g., in Fig. 4 the Eq. 2 results, which come from a statistically correct treatment, still give a *χ*^2^ value of 39.4 in the weighted average, as compared with the expected *ν* = 15. Similarly, the *χ*^2^ values for the fits in Fig. 6 are both about 25. Excessively large *χ*^2^ values can come from inadequacies in the fit model or from sources of variability other than random noise in the data, as must be responsible for the unphysical decline in *E* with dataset in Fig. 6. In fact, Spiess and I made just this argument in suggesting that excess ensemble dispersion might stem from imprecision in the volumetric methods used to prepare samples [7].

## Methods

The least-squares (LS) and Monte Carlo (MC) calculations employed procedures similar to those described in several previous works on qPCR analysis [7, 10, 15, 18]. For the former, the KaleidaGraph program and FORTRAN codes were employed. FORTRAN codes were used for the MC simulations, with typically 4 × 10^4^ data sets in a run. *C*_*q*_ estimates were obtained from the data of PGDW using the LL7 model as described in [7]; although these data have been baselined, a sloping baseline was included in the fit model to compensate for possible systematic errors from this baselining [10].

It is instructive to note that some correlated data, like differences obtained from the fluorescence data shown in Fig. 1, *can* be analyzed by LS methods that take the correlation into account. Differences can be represented by a linear transformation of the original data, with the transformation matrix **L** having mostly zeroes, with one value of + 1 and one of − 1 in each row [19, 20]. If the original data are independent and of constant uncertainty, then the difference values must be weighted by a matrix **W** = (**L L**^T^)^− 1^. In order to obtain this inverse matrix, the determinant of (**L L**^T^) must be nonzero. When this procedure is tried in the present case, this determinant = 0 when the number of differences exceeds 8 — a clear “tilt” from the attempt to exceed the information content in the original data. However, the correlated procedure succeeds when any 8 differences that sample all 9 points are fitted to Eq. 3, while a single one of the 9 values is fitted to Eq. 2. And it yields results for the parameters and their SEs that are identical to those from the direct fit of all points to Eq. 2. (There is no correlation problem if no data point is used more than once in generating the pairs; but that would mean only 4 pairs for 9 points, hence reduced precision.)

## Supplementary information

**Additional file 1: Figure S1.** Histogrammed results for *E* estimated using pairs ratio method of Eq. 4, with fits to the normal distribution. **Table S1.***C*_*q*_ estimates for all 96 reactions from [11] using methods from [7]. **Tables S2-S4.** Results obtained analyzing data from [11] using Eqs. 2–4. **Figure S2**. Variance analysis of Cy0-calibration-based *E* estimates and their SEs. **Figure S3**. Fitted normal distributions for histogrammed normalized residuals from weighted averages of pairwise ratio *E* estimates for two of 16 replicate datasets.

## Data Availability

All data analyzed here are either available from already published sources or were generated in Monte Carlo simulations. All results are displayed in the paper and provided in numerical tables in the Supplementary Information.

## Abbreviations

qPCR: Quantitative polymerase chain reaction
*y* and *y*_0_: Fluorescence signal above baseline at cycle *x* and at cycle 0
AE and *E*: Amplification efficiency
*C*_*q*_: Quantification cycle
*N*_0_: Initial number of target molecules in sample
FDM and SDM: Cycles where *y* reaches its maximal first and second derivatives, respectively
Cy0: Intersection of a straight line tangent to the curve at the FDM with the baseline-corrected *x*-axis
*C*_*r*_: Threshold defined as a fraction of plateau level *y*_max_
LS: Least squares
*χ*^2^: Chi-square
*w*_*i*_: Statistical weight for *i*th data point
*σ*^2^ and *σ*: Variance and standard deviation
“Chisq” in KaleidaGraph fit results: *χ*^2^ when *w*_*i*_ = 1/*σ*_*i*_^2^), sum of squared residuals otherwise
*ν*: Statistical degrees of freedom, = # of data points - # of adjustable parameters
SD: Standard deviation
SE: Parameter standard error
SI: Supplementary Information
