# Supplementary material for: Critique of the pairwise method for estimating qPCR amplification efficiency: beware of correlated data!
Source: BMC Bioinformatics. 2020 Jul 8;21:291. doi: 10.1186/s12859-020-03604-4 (PMC7346608; doi:10.1186/s12859-020-03604-4)
Supplement: Supplementary file 1 — Additional file 1: Figure S1. Histogrammed results for E estimated using pairs ratio method of Eq. 4, with fits to the normal distribution. Table S1.Cq estimates for all 96 reactions from [11] using methods from [7]. Tables S2-S4. Results obtained analyzing data from [11] using Eqs. 2–4. Figure S2. Variance analysis of Cy0-calibration-based E estimates and their SEs. Figure S3. Fitted normal distributions for histogrammed normalized residuals from weighted averages of pairwise ratio E estimates for two of 16 replicate datasets. [file 12859_2020_3604_MOESM1_ESM.pdf]

Critique of the pairwise method for estimating qPCR amplification efficiency: Beware of correlated data!

Supplementary Information

Joel Tellinghuisen, Department of Chemistry, Vanderbilt University

Nashville, Tennessee, USA 37235

I present here additional information about the computations described in the paper, along with tables of numerical results.

Monte Carlo Simulations

Figure 3 in the paper compares results from the MC simulations for the 9-point model, as analyzed using the direct fit approach of Eq. 2, and by the differences method of Eq. 3. Results obtained using Eq. 4, as used by Panina, *et al.*, are compared with those from the direct analysis here in Fig. S1. Both the weighted Eq. 4 analysis and the direct fit produce distributions that are normal at this level of scrutiny (4×10^4 simulations). The precision loss by Eq. 4 (weighted) is only 5%, but there is a statistically significant negative bias. Unweighted use of Eq. 4 gives nonnormal estimates, significant loss of precision, and comparable bias, now positive.

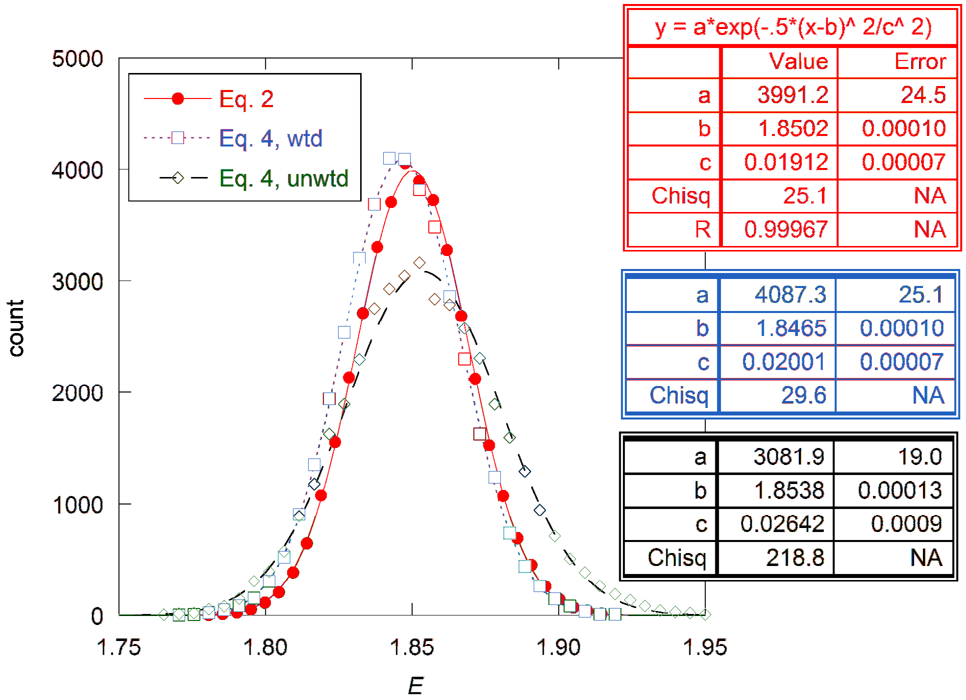

Fig. S1 Histogrammed *E* values from 4×10^4 simulations of the 9-point model described in text, analyzed directly by Eq. 2 and by the pairwise ratios method of Eq. 4, both weighted and unweighted. Fit results are for the weighted normal distribution. ($c = \sigma$.)

The problem with using Eq. 4 is not the nominal precision loss. It is that without the benefit of the MC simulations, the user could be deceived by the parametric SEs from individual datasets into concluding that the precision is much better — in this case more than a factor of 2 better, at 0.0097. This is precisely the problem with correlated data: They do not yield statistics that correctly predict results for other equivalent datasets. To be perfectly clear about the difference here: Random error is added *just* to the original intensity values. If random error were added to the differences, the results *would* agree with predictions. But of course it is the original data that are subject to random error, not the differences.

Estimating C_q from qPCR growth profiles

In ref [7] in the paper, Spiess and I described methods that can yield improved estimates of C_q, defined five ways. Four of these are compared with those from PGDW in Fig. 5 in the paper, where the χ^2 values for calibration fitting are smaller for all four. The method involves fitting the profile data to a 4-parameter log-logistic function plus a background, which was taken to be linear for these data. Also, best results are obtained for fitting just 20-30 cycles around the FDM rather than the full curve. The results shown in Fig. 5 were obtained fitting 26 points, but slightly better results were obtained for 28 points, and these were used to obtain the calibration results in Fig. 6. The C_q results for all 96 reactions from PGDW are given here in Table S1.

Table S1. Estimated C_q values for data from ref. [11]. N1 & N2 give the range of fitted cycles. `C5` is the location parameter in the log-logistic fit function. `#iters` is the number of iterations to convergence on the NLS fit. `RCS` is the reduced chi-square, χ^2 divided by (#pts – 6).

1 Run Panina data -- June 13, 2019.

Dil.	Set	#iters	N1	N2	#pts	RCS	C5	SDC5	FDM	SDM	THR	CY0
1	1	5	5	32	28	9.3928	16.7852	.5439	18.8489	16.3058	16.6406	15.9710
1	2	6	4	31	28	8.5726	15.8178	.7079	18.3888	15.7986	16.2450	15.5256
1	3	6	5	32	28	10.644	16.0739	.7240	18.7055	16.0175	16.4845	15.7354
1	4	12	5	32	28	7.6093	15.3245	.8078	18.6556	16.0060	16.5687	15.7979
1	5	6	4	31	28	5.4788	17.3000	.3845	18.8572	16.1958	16.4365	15.7704
1	6	6	4	31	28	3.7350	16.0134	.4829	18.7356	16.0063	16.4957	15.7291
1	7	5	4	31	28	9.3206	16.3824	.6577	18.7065	16.0632	16.4683	15.7506
1	8	5	5	32	28	6.5178	16.7928	.4794	18.7645	16.1849	16.5089	15.8339
1	9	9	5	32	28	9.5068	15.8258	.7045	18.6712	16.1569	16.6247	15.9180
1	10	5	3	30	28	11.421	17.0551	.6416	18.7712	16.1003	16.3831	15.7002
1	11	4	4	31	28	3.9593	16.3934	.4870	18.6684	15.8911	16.3134	15.5572
1	12	6	4	31	28	7.9074	15.7785	.7679	18.6846	15.8509	16.3933	15.5832
1	13	5	5	32	28	9.9893	16.5701	.7150	19.0273	16.1886	16.6464	15.8645
1	14	5	5	32	28	6.1649	16.6210	.5334	18.9660	16.1489	16.5834	15.8143
1	15	10	5	32	28	4.5403	15.5744	.6738	18.7766	16.0619	16.6188	15.8340
1	16	5	5	32	28	9.9518	16.4112	.6842	18.7960	16.1367	16.5533	15.8282
2	1	6	6	33	28	7.6269	17.2208	.6155	20.0198	17.3401	17.8003	17.0599
2	2	6	5	32	28	13.268	17.8667	.8647	20.0052	17.4722	17.7930	17.1336
2	3	35	6	33	28	7.0898	15.6156	1.0321	19.8410	17.3098	17.9187	17.1677
2	4	40	5	32	28	8.8287	15.3861	1.1492	19.7333	17.0841	17.7346	16.9405
2	5	7	6	33	28	12.569	16.4208	.9121	19.8458	17.1511	17.7078	16.9322
2	6	5	6	33	28	11.776	17.7416	.6763	19.9412	17.2113	17.5750	16.8539
2	7	6	6	33	28	9.1681	16.8114	.8716	19.9291	17.2154	17.7322	16.9649
2	8	6	5	32	28	10.935	17.4455	.7434	19.9136	17.2822	17.6811	16.9721
2	9	7	6	33	28	15.856	16.9238	.9473	19.9236	17.2345	17.7295	16.9752
2	10	5	6	33	28	9.6338	17.4500	.6255	19.8352	17.2614	17.6383	16.9502
2	11	7	6	33	28	8.6433	16.9585	.7827	20.1108	17.3169	17.8502	17.0584
2	12	6	6	33	28	10.647	16.8332	.8851	19.9016	17.2257	17.7287	16.9751
2	13	7	6	33	28	9.4642	17.0679	.8622	20.3362	17.3944	17.9695	17.1282

2	14	79	6	33	28	14.179	14.6124	1.8023	19.9416	17.2238	17.9677	17.1281
2	15	8	6	33	28	6.2761	15.7858	1.0218	19.9736	17.3256	17.9557	17.1692
2	16	7	5	32	28	11.175	16.4582	.9877	19.9047	17.3107	17.8485	17.1042
3	1	30	7	33	27	11.780	17.0382	1.1924	21.0650	18.4040	18.9993	18.2244
3	2	7	7	33	27	13.221	18.1393	.9620	21.1856	18.3558	18.8549	18.0690
3	3	30	7	33	27	9.3084	16.8821	1.1586	21.0527	18.2609	18.9003	18.0788
3	4	8	6	33	28	13.138	17.4984	1.1175	21.1137	18.2956	18.8743	18.0656
3	5	7	7	33	27	10.007	17.6880	.9169	21.1353	18.3098	18.8673	18.0636
3	6	7	7	33	27	12.395	17.7383	.9844	21.0894	18.3593	18.8865	18.1158
3	7	7	7	33	27	7.3283	17.5349	.8508	21.0737	18.2904	18.8531	18.0579
3	8	6	7	33	27	10.019	18.4489	.7838	21.1351	18.4282	18.8495	18.1186
3	9	25	7	33	27	12.211	17.1248	1.1869	21.0226	18.2825	18.8812	18.0853
3	10	28	7	33	27	8.9471	17.0550	.9972	21.0407	18.3733	18.9661	18.1903
3	11	9	7	33	27	9.4803	16.3423	1.5264	21.2470	18.3102	19.0524	18.1629
3	12	8	6	33	28	8.7419	17.3334	1.0806	21.3052	18.5059	19.1198	18.3053
3	13	9	7	33	27	6.8269	16.3301	1.2309	21.3037	18.3464	19.0989	18.2012
3	14	8	7	33	27	8.3359	17.2300	.9696	21.3254	18.4978	19.1313	18.3037
3	15	6	7	33	27	5.9943	17.6751	.8326	21.0325	18.2136	18.7598	17.9609
3	16	23	7	33	27	11.670	17.3570	1.2432	21.1126	18.4426	19.0088	18.2409
4	1	7	9	33	25	4.9884	18.3260	.9079	22.2935	19.6325	20.1999	19.4375
4	2	6	6	33	28	4.3052	19.6995	.5772	22.4540	19.6212	20.0453	19.2868
4	3	7	8	33	26	7.6889	19.1214	.8977	22.3984	19.6120	20.1127	19.3407
4	4	7	8	33	26	6.1713	19.2229	.8075	22.4784	19.5764	20.0919	19.2872
4	5	7	8	33	26	9.4451	18.8833	.9785	22.3754	19.6240	20.1489	19.3778
4	6	8	8	33	26	9.6731	19.2505	.9661	22.3820	19.6793	20.1458	19.4050
4	7	7	8	33	26	6.7579	20.3026	.6020	22.5038	19.7737	20.0785	19.3836
4	8	6	7	33	27	5.7064	18.6442	.7948	22.2472	19.4725	20.0184	19.2346
4	9	8	8	33	26	7.7611	18.7987	.9040	22.3830	19.5895	20.1339	19.3464
4	10	7	8	33	26	10.616	19.1226	.9597	22.2923	19.6086	20.0793	19.3415
4	11	12	8	33	26	6.6808	17.6022	1.3123	22.4894	19.5537	20.2686	19.3930
4	12	8	8	33	26	10.988	18.5849	1.3184	22.4376	19.6727	20.2442	19.4554
4	13	7	8	33	26	4.1752	18.0436	.9049	22.4978	19.4877	20.1766	19.2903
4	14	7	8	33	26	11.908	17.8095	1.6230	22.5857	19.6357	20.3418	19.4656
4	15	28	9	33	25	12.295	18.4951	1.4490	22.4273	19.6204	20.2095	19.4048
4	16	299	9	33	25	-1.0000	13.7103	1.4490	22.2786	19.5724	20.4073	19.5537
5	1	5	9	33	25	13.344	20.9287	.9627	23.6152	20.8583	21.2376	20.5148
5	2	5	8	33	26	8.2784	21.4782	.6642	23.6321	20.8905	21.1640	20.4793
5	3	4	9	33	25	8.5258	21.3164	.7852	23.6949	20.9315	21.2523	20.5471
5	4	6	10	33	24	16.972	20.6079	1.3720	23.7200	20.8855	21.3440	20.5775
5	5	5	9	33	25	7.2694	20.7217	.8052	23.6005	20.7465	21.1719	20.4110
5	6	6	9	33	25	6.7208	20.6446	.8095	23.6052	20.8206	21.2501	20.5046
5	7	12	9	33	25	10.027	20.0575	1.0081	23.2715	20.5337	21.0017	20.2543
5	8	68	10	33	24	8.6707	18.6934	1.5433	23.3947	20.7553	21.3723	20.6037
5	9	4	9	33	25	6.3727	21.2115	.6442	23.6621	20.8996	21.2351	20.5252
5	10	5	10	33	24	10.101	20.8722	.9382	23.5951	20.9378	21.3121	20.6146
5	11	7	9	33	25	6.4699	19.9142	1.0508	23.6745	20.7545	21.3201	20.4988
5	12	6	9	33	25	5.2075	20.3388	.8226	23.7181	20.7730	21.2890	20.4774
5	13	71	9	33	25	6.3916	18.7172	1.3686	23.6230	20.7808	21.4557	20.6198
5	14	8	10	33	24	10.027	19.4107	1.4673	23.9028	20.9612	21.6125	20.7600
5	15	34	10	33	24	6.6234	19.4869	1.2149	23.4539	20.7503	21.3066	20.5407
5	16	70	9	33	25	12.294	18.6427	1.9941	23.5197	20.7254	21.3889	20.5681
6	1	6	11	33	23	12.688	22.0737	1.0797	24.7557	22.0103	22.3678	21.6573
6	2	74	10	33	24	15.559	19.6814	2.5053	24.3996	21.7563	22.3617	21.5984
6	3	6	10	33	24	4.8539	22.2796	.7315	24.9312	22.0614	22.4236	21.6825
6	4	5	10	33	24	12.087	23.0598	.8843	24.8382	22.1384	22.2992	21.6640
6	5	7	10	33	24	7.6394	22.1073	.8816	24.6419	21.8543	22.1908	21.4769
6	6	6	10	33	24	13.429	22.1578	1.0289	24.6601	22.0091	22.3261	21.6507
6	7	6	9	33	25	9.2602	23.0736	.7175	24.8007	22.0709	22.2212	21.5826
6	8	5	10	33	24	11.746	23.0558	.7606	24.7743	22.1668	22.3086	21.7010
6	9	8	10	33	24	8.8704	20.8752	1.2250	24.6574	21.9909	22.5018	21.7620
6	10	6	11	33	23	10.985	22.2148	.8627	24.6367	22.1411	22.4307	21.8005
6	11	6	11	33	23	5.7628	22.4188	.8583	25.0174	22.1199	22.4735	21.7289
6	12	5	11	33	23	5.9362	23.1578	.6041	24.9054	22.2210	22.3720	21.7438
6	13	5	11	33	23	8.1411	22.7523	.9277	25.0895	22.1907	22.4906	21.7633
6	14	6	9	33	25	14.336	23.5301	.8889	25.1629	22.4282	22.5474	21.9187
6	15	7	11	33	23	6.0524	21.3960	1.0323	24.7730	22.0604	22.5239	21.7879
6	16	11	11	33	23	13.057	22.3021	1.2622	25.5365	22.5497	23.0161	22.2151

Estimating *E* by pairwise methods

Results for analysis of the 16 replicate datasets by Eqs. 2-4 are presented in Tables S2-S4.

Table S2. Results from analysis of data from [11] using Eq. 2. Results are ordered as A1-6, A7-12, B1-6, … H7-12. The number of differences analyzed for each set is the same as given in Table S3.

Y0	SE	E	SE	s_y^2
5.30330E-03	1.15809E-03	1.77498E+00	1.88059E-02	1.11248E+02
4.76390E-03	1.96314E-03	1.77610E+00	3.57371E-02	3.36285E+02
5.98552E-03	9.58350E-04	1.76192E+00	1.35004E-02	6.44547E+01
5.83466E-03	8.70279E-04	1.76461E+00	1.25902E-02	5.65896E+01
5.91630E-03	6.07848E-04	1.77127E+00	8.78686E-03	3.09130E+01
4.63665E-03	6.00433E-04	1.78948E+00	1.11234E-02	4.50340E+01
3.18663E-03	5.16272E-04	1.82350E+00	1.43689E-02	6.06855E+01
4.98656E-03	6.06858E-04	1.78203E+00	1.04353E-02	3.91143E+01
5.20159E-03	3.68158E-04	1.78232E+00	6.21435E-03	1.22121E+01
4.91855E-03	7.51945E-04	1.78496E+00	1.31094E-02	6.27018E+01
5.94004E-03	9.74526E-04	1.75699E+00	1.38007E-02	5.98791E+01
5.55975E-03	8.61753E-04	1.76602E+00	1.30894E-02	5.72580E+01
4.45983E-03	4.93879E-04	1.78203E+00	9.39328E-03	2.66079E+01
6.84292E-03	7.32664E-04	1.74532E+00	8.98222E-03	2.58502E+01
2.88372E-03	4.82212E-04	1.81331E+00	1.43274E-02	4.85800E+01
5.75604E-03	9.58512E-04	1.75808E+00	1.40115E-02	5.89280E+01

Table S3. Results from analysis of data from [11] using Eq. 3.

Y0	SE	E	SE	s_y^2	# diffs
4.57536E-03	3.26724E-04	1.78365E+00	5.78199E-03	1.99862E+02	2.76000E+02
2.92683E-03	3.75268E-04	1.80566E+00	1.07390E-02	4.80254E+02	2.76000E+02
5.24500E-03	2.76875E-04	1.76966E+00	4.15326E-03	1.13695E+02	2.76000E+02
5.00423E-03	2.43039E-04	1.77356E+00	3.81926E-03	9.46407E+01	2.76000E+02
5.41571E-03	1.76288E-04	1.77619E+00	2.59207E-03	5.31061E+01	2.76000E+02
4.10199E-03	1.75623E-04	1.79649E+00	3.40958E-03	7.62774E+01	2.53000E+02
2.63478E-03	1.33541E-04	1.83444E+00	4.20191E-03	9.14717E+01	2.53000E+02
4.61038E-03	1.88405E-04	1.78647E+00	3.24504E-03	7.10077E+01	2.53000E+02
5.10411E-03	1.15349E-04	1.78329E+00	1.86471E-03	2.31309E+01	2.53000E+02
4.46035E-03	2.26849E-04	1.79042E+00	4.06267E-03	1.13119E+02	2.53000E+02
4.69722E-03	2.28090E-04	1.77120E+00	3.85982E-03	8.53660E+01	3.00000E+02
4.87538E-03	2.50539E-04	1.77379E+00	4.04165E-03	1.01072E+02	2.76000E+02
3.80298E-03	1.32771E-04	1.79153E+00	2.74973E-03	3.87938E+01	2.53000E+02
6.65795E-03	2.45904E-04	1.74682E+00	2.86272E-03	4.91966E+01	2.31000E+02
2.15447E-03	1.06140E-04	1.83156E+00	3.98038E-03	5.65090E+01	2.31000E+02
5.00999E-03	2.69534E-04	1.76637E+00	4.26229E-03	1.02863E+02	2.76000E+02

Table S4. Results from weighted analysis of data from [11] using Eq. 4. The RCS values are based on the assumption that $s_y^2 = 50$.

E	SE	RCS	# pairs
1.78380E+00	4.82289E-03	1.02779E+00	2.54000E+02
1.75882E+00	7.84807E-03	3.03537E+00	2.54000E+02
1.76479E+00	3.86218E-03	7.89263E-01	2.57000E+02
1.77109E+00	3.59003E-03	6.59082E-01	2.56000E+02
1.77742E+00	2.56263E-03	3.49246E-01	2.55000E+02
1.79053E+00	3.32169E-03	5.15560E-01	2.34000E+02
1.83310E+00	4.64985E-03	7.71649E-01	2.33000E+02
1.78484E+00	3.17454E-03	4.76038E-01	2.35000E+02
1.78168E+00	2.09220E-03	1.75478E-01	2.33000E+02
1.78557E+00	3.80047E-03	6.69946E-01	2.32000E+02
1.76095E+00	3.20282E-03	5.48996E-01	2.77000E+02
1.76887E+00	3.65108E-03	6.72276E-01	2.56000E+02
1.78711E+00	2.99359E-03	3.82470E-01	2.35000E+02
1.74593E+00	2.51552E-03	2.87044E-01	2.15000E+02
1.81153E+00	4.09816E-03	6.09460E-01	2.11000E+02
1.75856E+00	3.42754E-03	5.89834E-01	2.55000E+02

Variance analysis of *E* estimates

In each of the statistically proper unweighted analyses — of C_q values by calibration fitting, or fitting of intensity data to Eq. 2 — the computations provide estimates of *E* and its SE. The latter must vary with replicate data set, from the random nature of data; and if the results are to be used in subsequent computations, as the fits of Figs. 4 and 6, the question arises: Should these SEs be used to weight the *E*s (as $1/\mathrm{SE}^2$)? With respect to Fig. 6, the answer was no, by the following analysis.

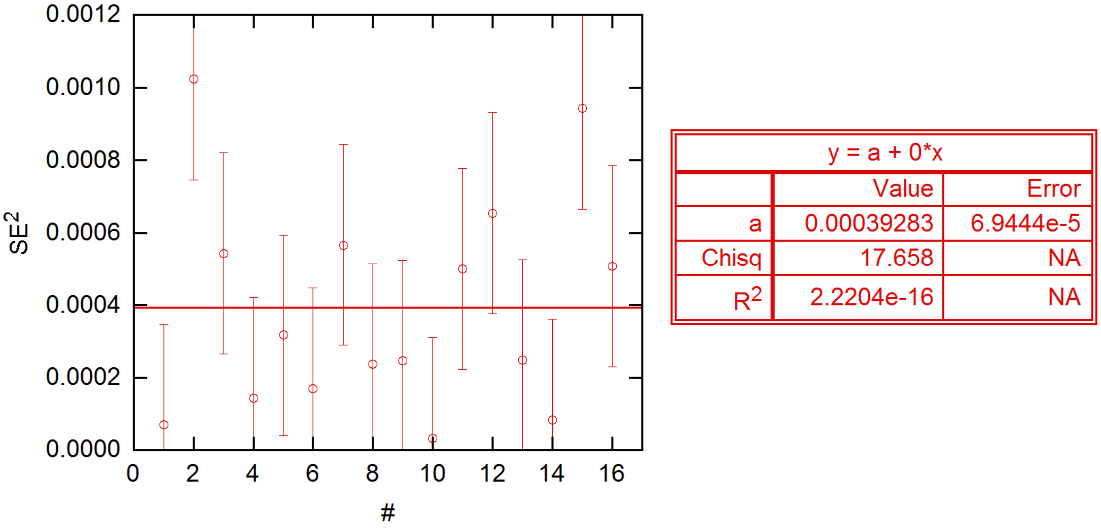

Fig. S2. Weighted fit of squared SE estimates for *E* from Cy0 calibration fits for the 16 replicate datasets. The illustrated error bars are $a \times (2/\nu)^{1/2} = a/2^{1/2}$. Weights are the reciprocals of this constant. (The unusual fit expression is needed to get KaleidaGraph to fit to a constant.)

Each C_q-calibration-based E is a result from fitting 6 C_q values to two parameters, so it has inherent relative uncertainty $(2\nu)^{-1/2} = 1/8^{1/2}$. Or equivalently, SE^2 has relative uncertainty $(2/4)^{1/2}$. Figure S2 shows the fit of the Cy0-based s_E^2 values from Fig. 6 to a constant (*i.e.*, weighted average). Under the assumption that this variance is constant, all values should have the same uncertainty, equal to $a/2^{1/2}$. The resulting $\chi^2 = 17.7$ is reasonably close to its expected value of 15.

Check of normal distribution for pairwise ratio estimates of *E*

I have examined the normalized residuals from weighted averages of the ratio-based (Eq. 4) pairwise estimates of E for 7 datasets: A1, A7, B1, D1, G7, and H1 (2 binnings). As noted in the paper, histograms of these are not adequately normal in most cases, but they are also not radically nonnormal. The RCS values from the weighted fits of the histogram data to the normal distribution range from 0.98 (D1) to 2.36 (A7) and translate into χ^2 probabilities from ~1% to 50%. Results are shown here for the two extreme cases.

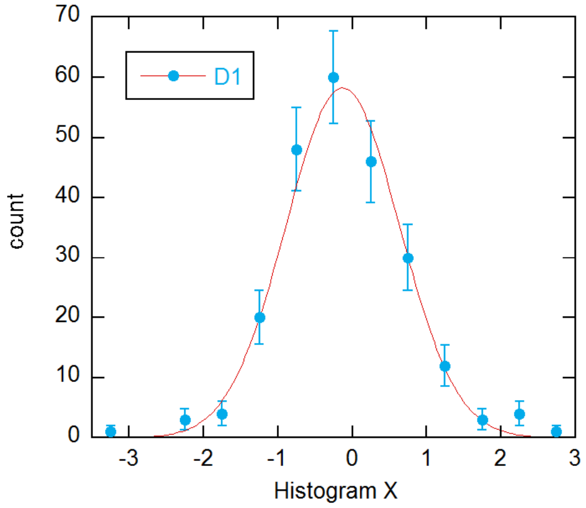

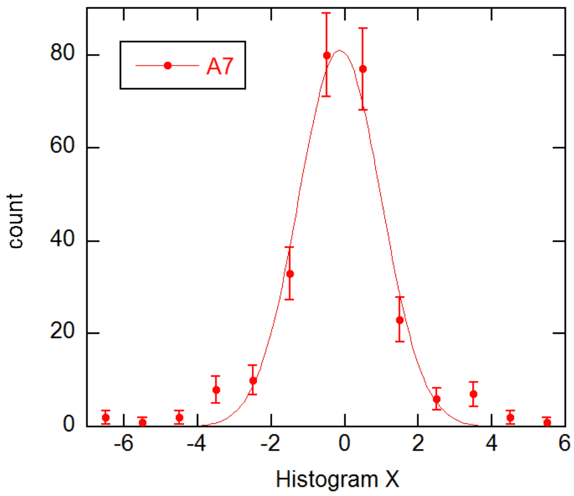

Fig. S3. Histograms of normalized residuals from weighted averages of Eq. 4-based E estimates for two of the 16 replicate datasets from [11]. The curves show weighted LS fits to the normal distribution and give $\chi^2 = 8.8$ for D1-6 and 23.6 for A7-12.
